# Topological model selection: a case-study in tumour-induced angiogenesis

**DOI:** 10.1093/bioinformatics/btag065

**Published:** 2026-03-12

**Authors:** Robert A McDonald, Helen M Byrne, Heather A Harrington, Thomas Thorne, Bernadette J Stolz

**Affiliations:** Mathematical Institute, University of Oxford, Radcliffe Observatory Quarter, Oxford OX2 6GG, United Kingdom; Mathematical Institute, University of Oxford, Radcliffe Observatory Quarter, Oxford OX2 6GG, United Kingdom; Nuffield Department of Medicine, Ludwig Institute for Cancer Research, Oxford OX3 7DQ, United Kingdom; Mathematical Institute, University of Oxford, Radcliffe Observatory Quarter, Oxford OX2 6GG, United Kingdom; Faculty of Mathematics, Technische Universitat Dresden, Dresden 01062, Germany; Centre for Systems Biology Dresden (CSBD), Dresden 01062, Germany; Max Planck Institute of Molecular Cell Biology and Genetics (MPI-CBG), Dresden 01307, Germany; Computer Science Research Centre, University of Surrey, Guildford GU2 7XH, United Kingdom; Department of Machine Learning and Systems Biology, Max Planck Institute of Biochemistry, Martinsried 82152, Germany; Munich Center for Machine Learning, Munich, 80538, Germany

## Abstract

**Motivation:**

Comparing mathematical models offers a means to evaluate competing scientific theories. However, exact methods of model calibration are not applicable to many probabilistic models which simulate high-dimensional spatio-temporal data. Approximate Bayesian Computation is a widely used method for parameter inference and model selection in such scenarios, and it may be combined with Topological Data Analysis to study models which simulate data with fine spatial structure.

**Results:**

We develop a flexible pipeline for parameter inference and model selection in spatio-temporal models. Our pipeline identifies topological summary statistics which quantify spatio-temporal data and uses them to approximate parameter and model posterior distributions. We validate our pipeline on models of tumour-induced angiogenesis, inferring four parameters in three established models and identifying the correct model in synthetic test-cases.

**Availability and implementation:**

Simulation code for all models, data analyses, parameter inference and model selection is available online at https://github.com/rmcdomaths/tms/ and archived at https://doi.org/10.5281/zenodo.17392787.

## 1 Introduction

Given multiple mathematical models which aim to reproduce the same biological data, determining which model and parameters give the best fit presents a theoretical and computational challenge. For example, spatio-temporal models often simulate complex high-dimensional data which is difficult to quantify and compare to observed data. Such models do not in general yield tractable likelihood functions, which significantly hinders the use of exact methods for parameter inference and model selection (Kirk *et al.* 2013).

Many mathematical models have been developed to study the mechanisms underlying tumour-induced angiogenesis (Scianna *et al.* 2013, Vilanova *et al.* 2017), a hallmark of cancer (Hanahan and Weinberg 2011). Tumour cells use chemical signals to stimulate the growth of new blood vessels from existing vasculature (Ferrara 2002), which provide a tumour mass with oxygen and nutrients that it requires to grow. However, instead of concise equations determining the growth of angiogenic networks, such models often comprise multiple agents and heterogeneous environments whose interactions depend non-deterministically on their spatial organization. Discrete models of tumour-induced angiogenesis, e.g. use multiple model rules and parameters to reproduce the branches, loops, and multiple components that characterize real vascular networks.

We use Topological Data Analysis (TDA), Approximate Bayesian Computation (ABC), and Random Forests (RFs) to develop a pipeline for parameter inference and model selection applicable to spatio-temporal models. TDA offers a toolkit of methods for quantifying spatial data (Ghrist 2008, Carlsson 2009, Edelsbrunner and Harer 2010). TDA has previously been used to study multi-agent temporal systems (Topaz *et al.* 2015, Bhaskar *et al.* 2019, Stolz *et al.* 2024) and was used in related work to compare models of insect locomotion (Ulmer *et al.* 2019) and pattern formation in zebrafish (Cleveland *et al.* 2023). ABC provides a statistical framework for using model simulations to approximate posterior distributions when likelihood functions are not available (Lintusaari *et al.* 2017). RFs are an ensemble estimation method from machine learning which have previously been combined with ABC to estimate parameter values (Raynal *et al.* 2019) and rank candidate models (Pudlo *et al.* 2016).

We begin by outlining three existing models of tumour-induced angiogenesis in which exact methods of parameter inference and model selection are not applicable. We show how TDA can be used to characterize spatial data simulated by the models and briefly describe methods from ABC and RF. We then present a three-step pipeline for parameter inference and model selection which we apply to the angiogenesis models. Commenting on the applicability of our pipeline to experimental data, we discuss how topological summaries may be used to evaluate a variety of modelling approaches in biology.

## 2 Model data and analysis

### 2.1 Angiogenesis models

Discrete models of tumour-induced angiogenesis simulate the movement of individual Endothelial Cells (ECs). Vascular Endothelial Growth Factors (VEGF) produced by tumour cells initiate a cascade of chemical reactions which drive ECs towards the tumour. Early models used the *snail-trail model* (Balding and McElwain 1985) in which tip ECs migrate up spatial gradients of VEGF and fibronectin, while stalk ECs proliferate in their path to produce a contiguous line of cells. When two separate trails of ECs meet they fuse together to form a loop, in a process known as anastomosis. A tip EC may also split into two tip ECs, which thereafter move independently. Migrating, branching and looping tip ECs eventually reach the tumour mass and the connecting trails of stalk ECs form a blood vessel network. Recent models reflect modern discoveries of cell mixing and phenotype switching (Stepanova *et al.* 2024), where ECs change type and overtake each other before forming a stable blood vessel network. Other models view ECs as a continuous population density rather than individual cells (Martinson *et al.* 2021) and account for blood flow and nutrient delivery when simulating vascular networks (Hormuth *et al.* 2021). We develop our pipeline of parameter inference and model selection on discrete angiogenesis models due to their simulation of finely resolved spatial data.

**Figure 1:**
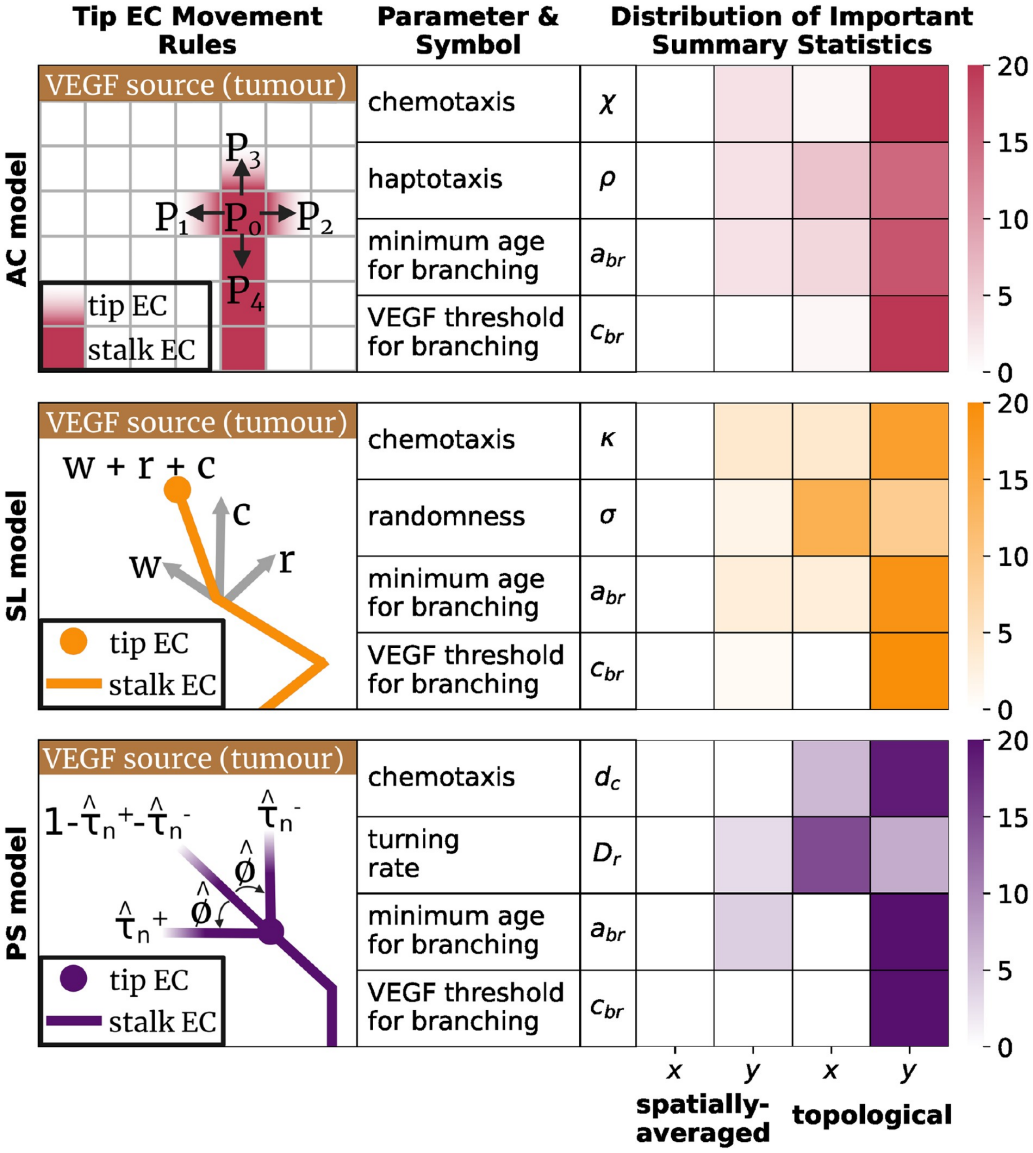
In the AC model (Anderson and Chaplain 1998), a tip endothelial cell (EC) makes one of five possible moves on a square lattice in each time-step according to probabilities $P_{0},P_{1},P_{2},P_{3},P_{4}$. A chemotaxis parameter χ biases movement probabilities in the direction of increasing VEGF concentration, and a haptotaxis parameter ρ biases moves in the direction of increasing fibronectin. In the SL model (Stokes *et al*. 1991), tip ECs move in any direction (off-lattice) with velocities modelled by a 2D stochastic differential equation. Parameters κ and σ determine how strongly an EC’s current velocity *w* is affected by the VEGF gradient *c*, and random variation *r*, respectively. The PS model (Plank and Sleeman 2004) assigns a constant speed to each tip EC and, at each time-step, rotates the angle that the velocity vector makes with the vertical. The probability ${\hat{\tau }}_{n}^{+}+{\hat{\tau }}_{n}^{-}$ that a tip EC turns by $\hat{\phi }$ is determined by a turning rate parameter $D_{r}$. A chemotaxis parameter $d_{c}$ biases turns that re-orient the EC’s direction towards the tumour. In all models, a tip EC may bifurcate into two ECs which thereafter move independently if its age exceeds the minimum age for branching parameter $a_{\text{br}}$ and the VEGF concentration at its location exceeds the VEGF threshold for branching parameter $c_{\text{br}}$. We show how many spatially averaged and topological summary statistics, computed in either the *x* or *y* co-ordinate direction, appear among the 100 most important summary statistics to the inference of each parameter.

The Anderson-Chaplain (AC) (Anderson and Chaplain 1998), Stokes-Lauffenberger (SL) (Stokes *et al.* 1991), and Plank-Sleeman (PS) (Plank and Sleeman 2004) models use the snail-trail model to simulate movement of individual ECs in a 2D, square domain. We assume that VEGF levels increase from the bottom of the domain to a tumour at the top, guiding ECs to move upwards. Each model initializes multiple distinct tip ECs along the bottom of the domain, simulating their trajectories according to model-specific movement rules. In each model, we choose four parameters that are likely to lead to measurable changes in simulated data and we attempt to infer their values. Figure 1 illustrates the movement rules and model parameters in each model. See Section 1, available as supplementary data at *Bioinformatics* online for full statements of each model and its parameters.

### 2.2 Data generation and analysis

Each angiogenesis model outlined in Section 2.1 (and described fully in Section 1, available as supplementary data at *Bioinformatics* online) simulates EC movement in a square domain. To summarize the spatial properties of each simulation, we overlay a regular grid onto the domain at the final timestep and compute a collection of *spatially-averaged* and *topological* summary statistics. For the *spatially-averaged* summary statistics, we compute the mean, standard deviation, minimum, maximum, range, and the 10th, 25th, 75th, and 90th percentiles of the *x* and *y* co-ordinates of EC locations in the grid. These were used in Nardini *et al.* (2021) to distinguish the AC model’s behaviour in different parameter regimes.

Persistent homology (PH) is a prominent method within Topological Data Analysis (TDA) (Ghrist 2008, Carlsson 2009, Edelsbrunner and Harer 2010) to quantify loops, branches, and connected components. Here, we require finer information than is provided by standard persistence; therefore, we use extended persistent homology (EPH) (Cohen-Steiner *et al.* 2009). We give an overview of PH and EPH, briefly indicating how EPH arises from PH and captures a greater range of spatial information within the angiogenesis data we simulate. See Section 2, available as supplementary data at *Bioinformatics* online for a full definition of EPH and a worked example.

To compute PH, a nested sequence of simplicial complexes, known as a filtration, is built on the data. Intuitively, a simplicial complex $\Sigma_{k}$ is a graph that includes nodes and edges as well as higher-order connections such as triangles or tetrahedra. A filtration must be carefully constructed such that each $\Sigma_{k}$ encodes the spatial properties of the underlying data at some spatial threshold defined by *k*. For example, Nardini *et al.* (2021) used a *sweeping-plane* filtration to analyse the AC model, where $\Sigma_{k}$ is constructed from those ECs which are a distance of *k* or more away from the tumour. Once a filtration has been chosen, one computes a sequence of F-vector spaces $H_{p}(\Sigma_{k})$ known as homology groups. We use the field F=Z/2Z, which is widely adopted in applications for its simplicity and interpretability. Homology groups quantify *p*-dimensional topological features in each $\Sigma_{k}$ [see, e.g. Otter *et al.* (2017)]. $H_{0}$ detects connected components, $H_{1}$ detects loops and, in general, $H_{p}$ detects *p*-dimensional voids. We consider dimensions 0 and 1 only, since voids of dimension 2 or higher do not appear in the angiogenesis data we simulate. Persistence pairs (b,d) are computed from the sequence of homology groups to quantify topological features in the filtration (Zomorodian and Carlsson 2005). A birth *b* corresponds to the index *k* in the filtration at which a topological feature first appears. A death *d* is either the index *k* at which the *p*-dimensional void it represents is filled in, or ∞ if the topological feature persists through the filtration. The difference d−b is known as the persistence of a topological feature. The interpretation of (b,d) in terms of the underlying data depends on the choice of filtration. In the sweeping-plane filtration used in Nardini *et al.* (2021), persistence pairs quantify connected components and loops in simulated vascular networks in terms of their distance from the tumour.

**Figure 2:**
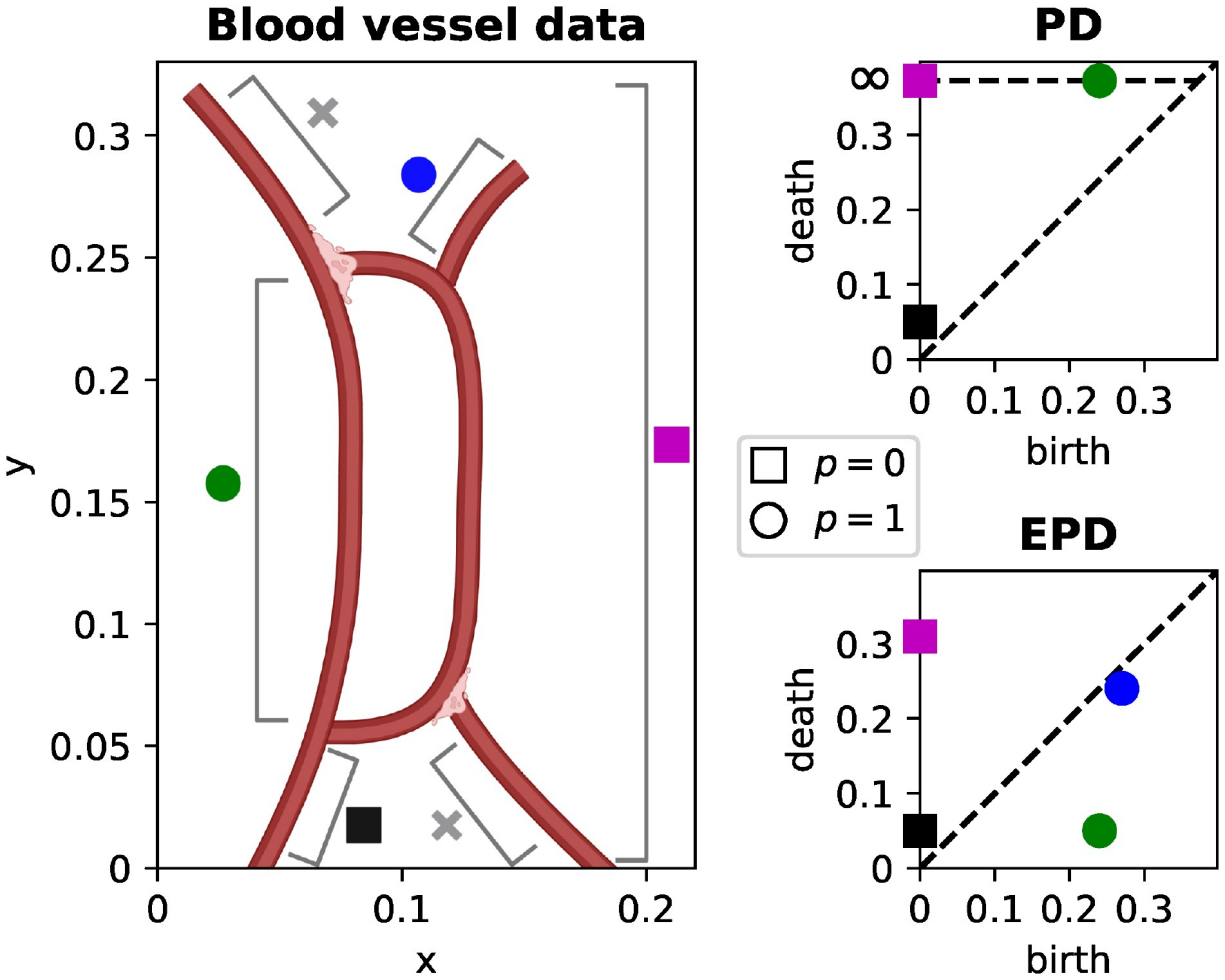
A persistence diagram (PD) and extended persistence diagram (EPD) for a simple blood vessel computed using the vertical sweeping-plane filtration. The PD points quantify the size and location of the small lower branch (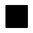), and the locations of the component (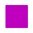) and loop (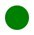). The EPD points quantify the location *and size* of all topological features quantified by the PD, in addition to the small upper branch (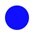). The branches (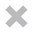) are not detected by PH or EPH with this sweeping-plane filtration.

In PH, some topological features typically persist throughout the entire filtration. In Fig. 2, e.g. a connected component appears at y=0 and a loop at y=0.25, and both persist for all values of *y* in the sweeping-plane filtration. The corresponding persistence pairs are therefore (0,∞) and (0.25,∞), which quantify limited location information and no size information about the topological features they represent. Furthermore, PH computes one PD in each dimension (p=0 and p=1 in this work), but we want to distinguish several different spatial structures (see Fig. 2 for examples).

We would like to quantify the size and location of different spatial features. EPH provides this information by appending *relative* homology groups (Edelsbrunner and Harer 2010) to the sequence of ordinary homology groups in PH. Topological features which persist through all ordinary homology groups will die in the relative homology groups, so persistence pairs computed from EPH are guaranteed to have finite persistence. Each EPH persistence pair then be classified as one of four types depending on where the birth and death appear in the sequence of ordinary and relative homology groups, which provides additional information about the corresponding topological features. See Section 2, available as supplementary data at *Bioinformatics* online for a formal definition of topological feature types in EPH and their interpretation in simulated angiogenesis data. Figure 2 compares persistence pairs computed from a simple blood vessel network using PH and EPH, illustrating the extra information provided by EPH. We compute two extended persistence diagrams (EPDs) for each angiogenesis dataset–using a vertical (y) and a horizontal (x) sweeping-plane filtration. We vectorize each EPD using Persistence Images (Adams *et al.* 2017) and persistence statistics (Ali *et al.* 2023), and our *topological* summary statistics are the concatenation of these vectors.

## 3 Materials and methods

### 3.1 Approximate Bayesian computation

ABC provides a statistical framework for using data to infer model parameters. Suppose a model uses parameters Θ to simulate data D according to some probability distribution p(D|Θ), called the *likelihood*. Parameter inference aims to determine the *posterior* distribution p(Θ|D), which is the probability that parameters Θ generated observed data D. Using previous experiments or assumptions about feasible parameter values, one may define a *prior* distribution p(Θ) representing knowledge of the parameter values before data has been observed. The likelihood, prior and posterior are related by Bayes’ rule,


$$p(\Theta |D)=\frac{p(D|\Theta )p(\Theta )}{p(D)},$$

where the evidence p(D) is the integral $\int_{\Theta }p(D|\Theta )p(\Theta )d\Theta$ over parameters Θ in the support of the prior. Although Bayes’ rule gives a closed formula for the posterior distribution, it is often impractical to use directly. The likelihood function p(D|Θ) may be too complicated to derive for probabilistic spatial models in which many datasets D may be simulated from the same parameters Θ. Instead, Bayes’ rule is used to derive Approximate Bayesian Computation (ABC) algorithms (Lintusaari *et al.* 2017) which allow sampling from the posterior when the likelihood and evidence are not known. ABC algorithms sample candidate parameters $\theta_{i}$ from the prior p(θ) and accept them if the distance $\nu (D_{i},D^{*})$ between simulated and observed data is less than some tolerance ϵ>0 for some distance function ν. If the tolerance ϵ is set to zero, then the distribution of accepted parameters is the posterior $p(\Theta |D^{*})$ (Frazier *et al.* 2018). However, it is often not appropriate or possible to seek an exact posterior distribution from observed data, since a model may rarely reproduce observed data $D^{*}$ exactly, and the observed data may be noisy. It is therefore advisable to choose ν and ϵ such that parameter values are accepted if they simulate data that is similar to observed data. The general form of such a distance function is $\nu (D^{*},D_{i})=∥X^{*}-X_{i}{∥}_{2}$ where $X_{i}$ and $X^{*}$ are vectors of *summary statistics* computed from model data $D_{i}$ and observed data $D^{*}$ respectively. Summary statistics aim to capture relevant properties of data as a low dimensional vector. As ϵ approaches 0, the distribution of parameters accepted by ABC algorithms approaches $p(\Theta |X^{*})$, which equals $p(\Theta |D^{*})$ if the summary statistics are sufficient for the model in question, or is a close approximation if the summary statistics are insufficient but informative (Joyce and Marjoram 2008).

### 3.2 Random Forests

Random Forests (RFs) (Breiman 2001) learn relationships between feature vectors and response variables. Training data comprising a collection of feature vectors $X_{i}\in X$ and corresponding response variables $y_{i}\in Y$ are used to train a RF, enabling it to predict the true response variable $y^{*}$ of an unseen feature vector $X^{*}$. Regression RFs are used when $y_{i}$ are continuous values and classification RFs are used when $y_{i}$ are discrete labels. Raynal *et al.* (2019) used a regression RF for parameter inference by using simulated data $D_{i}$ to learn the relationship between summary statistics $X_{i}$ and parameter values $y_{i}=\theta_{i}$. Given summary statistics $X^{*}$ of unseen data $D^{*}$, the prediction $RF(X^{*})$ predicts the true parameter value $\theta^{*}$. In addition to predicting unseen feature vectors, a trained RF provides useful information about the training data. The out-of-bag prediction $RF_{\text{oob}}(X_{i})$ estimates the (known) response variable $y_{i}$ using pairs from the training data other than $\left(X_{i},y_{i}\right)$. The out-of-bag error rate $p(RF(X_{i})\neq y_{i})$ then gives a (unbiased) measure of how well the relationship between $X_{i}$ and $y_{i}$ is captured by the rest of the training data. A trained RF also gives a measure of the *importance* of each co-variate *j* within feature vectors $X_{i}=(X_{i}^{0},\ldots ,X_{i}^{j},\ldots ,X_{i}^{n_{f}})$ to the problem of predicting response variable $y_{i}$. Intuitively, important features are those whose values within $X_{i}$ and $X_{i}^{\prime }$ differ when $y_{i}$ and $y_{i}^{\prime }$ do, and which are hence useful in learning the relationship between training data X and Y.

### 3.3 Model selection

Given observed data $D^{*}$, the model posterior $p(m|D^{*})$ gives the probability that models $m=m_{i}$ generated $D^{*}$. ABC algorithms rely on the approximation $p(\Theta |X^{*})\approx p(\Theta |D^{*})$, which holds as long as the vector $X_{i}$ carries a similar amount of information about the parameter value $\theta_{i}$ as the simulated data $D_{i}$ itself. However, the information loss suffered by a collection of summary statistics may vary between models (Robert *et al.* 2011), so it is inadvisable simply to infer $m_{i}$ as a (discrete) parameter using an ABC algorithm. Pudlo *et al.* (2016) instead used two RFs to approximate $p(m|D^{*})$. A classification RF learns the relationship between simulated data $X_{i}$ and model label $y_{i}=m_{i}$ and gives a prediction $RF(X^{*})$ of the model $m^{*}$ which generated unseen data $D^{*}$. A regression RF is then trained to learn the relationship between $X_{i}$ and $p(RF_{\text{oob}}(X_{i})\neq m_{i})$–the out-of-bag error rate of the classification RF. The regression RF is then used to estimate posterior probability $p(m=m^{*}|D^{*})$ as $1-p(RF(X^{*})\neq m^{*})$.

## 4 Spatial parameter inference and model selection

Given observed data $D^{*}$, we wish to approximate the parameter posterior $p(\Theta |D^{*})$ for candidate models $m=m_{1},m_{2},\ldots$ and the model posterior $p(m|D^{*})$. Informative summary statistics may be used to infer parameter values using ABC, but uninformative or poorly scaled summary statistics may misrepresent the difference between datasets generated by similar parameters (Blum *et al.* 2013). We therefore seek a collection of summary statistics that quantify simulated data and, in particular, quantify how simulated data changes when different model rules and parameters are used to generate it. We use informative summary statistics to approximate parameter and model posteriors in a three-step pipeline. We test this pipeline on toy models in Section 4, available as supplementary data at *Bioinformatics* online and apply it to the three angiogenesis models in Section 5.

**Step 1: Identify informative summary statistics** 

We use RFs to find a small subset of summary statistics to be used in ABC. To generate training data, we draw parameter values $\theta_{i}$ from the prior distribution p(Θ) for each parameter in each model, simulate model data $D_{i}$, and compute spatially averaged and topological summary statistics $X_{i}$ from the final simulated time-step. We train regression RFs to learn the relationship between summary statistics $X_{i}$ and parameter values $\theta_{i}$–one RF for each parameter in each model. We then rank the spatially averaged and topological summary statistics by their importance according to the RF (see Section 3.2). In each RF, feature importance decreases exponentially [as in Raynal *et al.* (2019)] and a small subset of summary statistics provides most of the predictive power of each RF. We select an equal number of informative summary statistics from each RF, collecting a total of $n_{s}=100$ for each model. See Section 3, available as supplementary data at *Bioinformatics* online for a full definition of RF feature importance and a discussion of how we choose $n_{s}$. RFs identify which summary statistics quantify the effect of each parameter on simulated data and allow us to omit those summary statistics which do not.

**Step 2: Fit each model to the observed data** 

We use the summary statistics identified by step 1 to define a distance function for use in ABC. We use $\nu (D^{*},D_{i})=∥x^{*}-x_{i}{∥}_{2}$, where $x_{i}$ is the vector $X_{i}$ restricted to the top $n_{s}$ summary statistics identified in step 1, $x^{*}$ is computed from observed data, and the distance is averaged over multiple instances of observed data. We scale each summary statistic by the largest absolute value of that summary statistic in the training data. We then use the ABC-SMC algorithm of Del Moral *et al.* (2012) to approximate $p(\Theta |D^{*})$ for each model. By using only those summary statistics which quantify the effect of parameter values on simulated data, we ensure ν is informative about the value of θ used to generate the observed data. Scaling ensures that each summary statistic contributes approximately equally to the distance function ν and limits the influence of poorly scaled summary statistics.

**Step 3: Approximate the model posterior** 

Using summary statistics which are informative for all models, we use two more RFs to estimate the model posterior. Following Pudlo *et al.* (2016), we train a classification RF to learn the relationship between (unscaled) summary statistics $X_{i}$ and model indices $m_{i}$ in the training data. We modify $X_{i}$ to contain only those summary statistics which appear among the $n_{s}$ most important summary statistics for all models. We then train a regression RF to learn the relationship between $X_{i}$ and $p(RF_{\text{oob}}(X_{i})\neq m_{i})$–the probability that the predicted model index is incorrect. The classification RF gives an estimate $RF(X^{*})$ of the model $m^{*}$ that generated the observed data $D^{*}$, and the regression RF is used to estimate $p(m=m^{*}|D^{*})$ as $1-p(RF_{\text{oob}}(X^{*})\neq m^{*})$. We choose the value of $n_{s}$ in step 2 large enough to ensure that some informative summary statistics are selected for all three models under consideration–we use these to approximate the model posterior.

## 5 Results

### 5.1 RFs find small subsets of informative summary statistics

We sample *n*=10,000 model parameters from uniform priors with ranges taken from existing literature, or by analysing each model’s data generation rules (See Section 1, available as supplementary data at *Bioinformatics* online for details). We select $n_{s}=100$ summary statistics for each model and report the type of summary statistics selected—spatially averaged or topological, computed in the horizontal or vertical direction—in Fig. 1. A mixture of spatially averaged and topological summary statistics are selected for each parameter, however there is a clear preference for topological summary statistics for each parameter. In general, summary statistics computed in the vertical direction are selected more often than those computed in the horizontal direction, which is unsurprising, since most parameters regulate the movement of ECs upwards. However, the randomness parameter σ of the SL model and the turning coefficient $D_{r}$ of the PS model are two exceptions. These parameters cause ECs to deviate from their upward trajectory, and their inference therefore relies on topological summary statistics computed in the horizontal direction. No spatially averaged summary statistics computed in the horizontal (*x*) direction are chosen, indicating that these measures are too coarse to distinguish different model simulations.

### 5.2 ABC-SMC infers four parameters for each model and reproduces observed data

**Figure 3:**
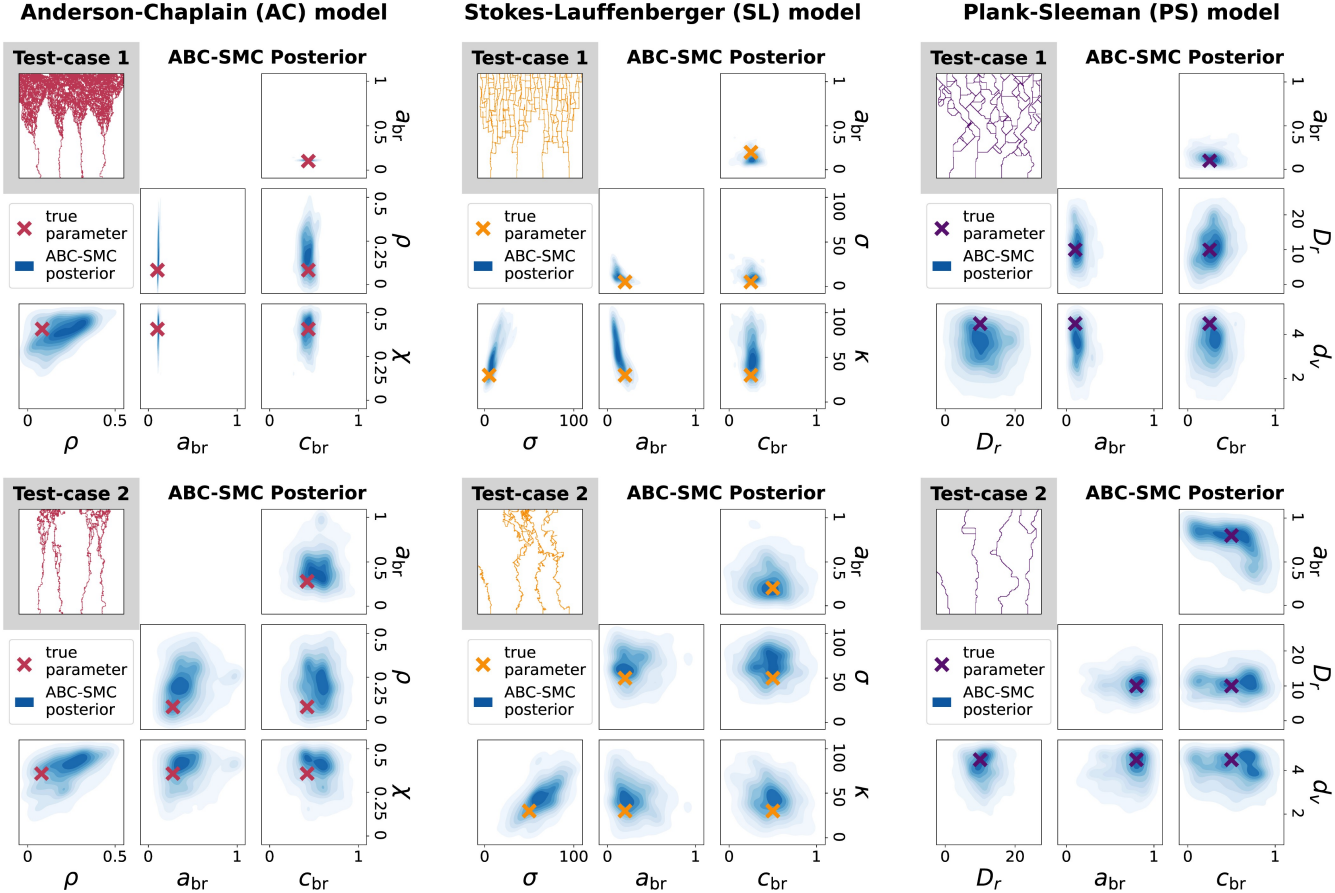
We infer the minimum age for branching ($a_{\text{br}}$) and VEGF threshold for branching ($c_{\text{br}}$) in each model, as well as chemotaxis and haptotaxis parameters (χ and ρ) in the AC model, chemotaxis and randomness parameters (κ and σ) in the SL model, and chemotaxis and turning rate parameters ($d_{c}$ and $D_{r}$) in the PS model. We simulate each model 10 times at known parameter values to generate two synthetic test-cases for each model, and show the final time-step of one such simulation. We then use steps 1–2 of Section 3 to approximate the parameter posterior $p(\Theta |D^{*})$ in each test-case. We project the approximate ABC-SMC posterior to each parameter pair and plot the resulting distributions (fitting a Gaussian kernel to the parameter values accepted in the final population of the ABC-SMC algorithm), along with the true parameter which generated the test-case.

We create two test-cases for each model by simulating data at known parameter values 10 times. Each test-case exhibits quantitatively different vascular networks that each model can produce. Following steps 1 and 2 of Section 4, we use ABC-SMC to infer four parameters for each model and show the resulting approximate posteriors in Fig. 3. In each test-case, the approximate posterior is unimodal and encompasses the true parameter, often close to its densest part.

### 5.3 Random Forests correctly select models

**Figure 4:**
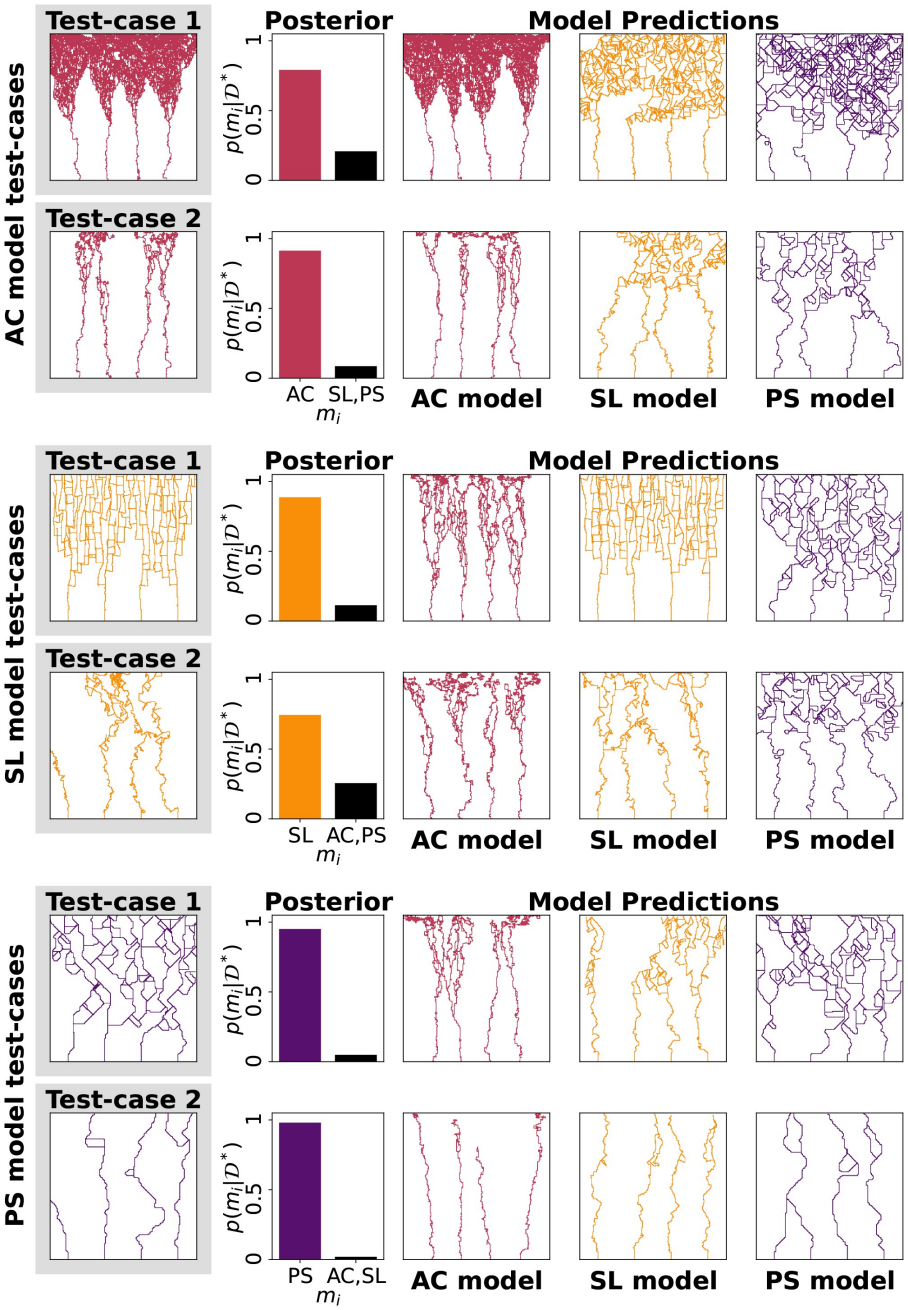
We approximate the model posterior $p(m|D^{*})$ using the six test-cases from Fig. 3, highlighting one example of $D^{*}$. For each test-case, we show one example of data simulated using an inferred parameter from each model’s approximate parameter posterior. Each ‘prediction’ shows an example of that model’s approximation of the true data generation process.

Using step 3 of Section 4, we approximate the model posterior for each of the six test-cases. Fig. 4 shows the resulting approximate model posteriors, which identify the correct model with high probability in each test-case. We simulate each model at a parameter value drawn from its approximate parameter posterior and show the resulting ‘prediction’, which is a sample from the model’s approximation of the true data generating process. Each model generates data that is visually similar to the observed data, however we can identify the true model in each test-case.

## 6 Discussion

Using Topological Data Analysis (TDA) and Approximate Bayesian Computation (ABC), we have developed a pipeline for parameter inference and model selection applicable to complex spatial models. In previous work, TDA characterized the effect of two parameters in the AC model (Nardini *et al.* 2021) and was combined with ABC to infer them (Thorne *et al.* 2022). Here we extend this work by identifying a subset of informative summary statistics from multiple topological filtrations and use them to infer four parameters in three angiogenesis models using ABC-SMC. We further show how RFs can be used with TDA to approximate model posteriors and compare candidate models.

While we validated our pipeline using synthetic data simulated from angiogenesis models, previous studies used *in vitro* data to inform model rules and parameters (Milde *et al.* 2013, Connor *et al.* 2015, Vergroesen *et al.* 2025). The present work therefore enhances previous model analysis, since ABC provides a statistical framework for learning parameters and evaluating models, and TDA provides a variety of filtrations and vectorizations which may be adapted to different spatial data. In future work we will apply our pipeline to real experimental data, which will take the place of the synthetic test-cases in Figs 3 and 4 wherein the true parameter values and correct model will not be known.

In this study, we considered three models in which the paths traced by tip ECs form a static blood vessel network. Topological features, therefore, evolve monotonically in time in these models, and computing EPH at the final timestep was sufficient to infer parameters. In reality, sustained proliferation and vessel remodelling, where vasculature continually evolves after it is laid down, is characteristic of tumour-induced angiogenesis (Farnsworth *et al.* 2014). Indeed, vascular renormalization, in which vessel-targetting agents prune small or inefficient blood vessels, is a theorized treatment strategy (Magnussen and Mills 2021) aiming to temporarily enhance perfusion of the tumour to increase the effectiveness of radiotherapy (Jain 2014). Topological invariants that account for time-evolving data (Xian *et al.* 2022), directed flow networks (Chaplin *et al.* 2024) and multiparameter filtrations (Vipond 2020) could quantify such structural changes over time and be used to calibrate more sophisticated models. Rather than fixing the duration of simulations in these cases, simulation time could be an extra parameter to be inferred by our pipeline.

Although we specialized our pipeline to discrete models of tumour-induced angiogenesis, its flexibility allows application to a range of spatio-temporal models. Any summary statistic which quantifies the desired properties of simulated data would be identified by the RF in step 1 if it captures the effect of changing model parameters. In future, we will use our pipeline to systematically compare continuum, cell-based, agent-based, and discrete models by their ability to reproduce observed data.

## Supplementary Material

btag065_Supplementary_Data

## Data Availability

All code is available at https://github.com/rmcdomaths/tms/ and archived at https://doi.org/10.5281/zenodo.17392787. Parts of figures 1 and 2 were created in Created in BioRender: Byrne, H. (2026) https://BioRender.com/vwzr7yj, https://BioRender.com/bql6i5f.
